# Vagus nerve stimulation for epilepsy: A narrative review of factors predictive of response

**DOI:** 10.1111/epi.18153

**Published:** 2024-10-16

**Authors:** Harry J. Clifford, Menaka P. Paranathala, Yujiang Wang, Rhys H. Thomas, Tiago da Silva Costa, John S. Duncan, Peter N. Taylor

**Affiliations:** ^1^ Computational Neurology Neurosicence and Psychiatry Lab, School of Computing Newcastle University Newcastle Upon Tyne UK; ^2^ Neurosciences Royal Victoria Infirmary Newcastle Upon Tyne UK; ^3^ Faculty of Medical Sciences Newcastle University Newcastle Upon Tyne UK; ^4^ UCL Queen Square Institute of Neurology London UK; ^5^ Northern Centre for Mood Disorders, Newcastle University, Cumbria, Northumberland Tyne and Wear NHS Foundation Trust Newcastle Upon Tyne UK; ^6^ National Institute for Health and Care Research, Newcastle Biomedical Research Centre Newcastle Upon Tyne UK

**Keywords:** EEG, MRI, prediction, Vagus nerve stimulation, VNS

## Abstract

Vagus nerve stimulation (VNS) is an established therapy for drug‐resistant epilepsy. However, there is a lack of reliable predictors of VNS response in clinical use. The identification of factors predictive of VNS response is important for patient selection and stratification as well as tailored stimulation programming. We conducted a narrative review of the existing literature on prognostic markers for VNS response using clinical, demographic, biochemical, and modality‐specific information such as from electroencephalography (EEG), magnetoencephalography, and magnetic resonance imaging (MRI). No individual marker demonstrated sufficient predictive power for individual patients, although several have been suggested, with some promising initial findings. Combining markers from underresearched modalities such as T1‐weighted MRI morphometrics and EEG may provide better strategies for treatment optimization.

**Key points**

- VNS is a safe and effective therapy for drug‐resistant epilepsy.
- Key brain areas implicated in VNS include the thalamus, amygdala, hippocampus, and insula, among others.
- Identifying markers of difference in response to VNS within DRE could explain heterogeneity in outcomes.
- Promising markers are present in imaging, neurophysiological, clinical, and biochemical data, but these are often underinvestigated.
- Validating potential markers in large multicenter cohorts will support their use in early response prediction for clinical care.

## 1 INTRODUCTION

The International League Against Epilepsy defines drug‐resistant epilepsy (DRE) as the “failure of adequate trials of two tolerated and appropriately chosen and used antiseizure medications (ASMs), as monotherapies or in combination, to achieve sustained seizure freedom.”^1^ DRE is associated with multiple negative short‐, medium‐, and long‐term biological and societal outcomes, including premature mortality.

If potentially curative neurosurgical resection is not viable, neuromodulation in the form of deep brain stimulation, responsive neurostimulation, or vagus nerve stimulation (VNS) may be offered.^2^ VNS is a palliative therapy and involves the insertion of an electrode around the left vagus nerve within the carotid sheath. A pulse generator is implanted, most commonly in the upper chest wall, and stimulator settings are programmed. Stimulation is hypothesized as having therapeutic effect via afferent stimulation of the nucleus tractus solitarius, which then projects to the locus coeruleus (LC) and beyond. Reported efficacy is approximately a 50% reduction in seizure frequency in approximately half of patients.^3^, ^4^, ^5^ Unlike other interventions for DRE, VNS is extracranial and therefore does not have the risks of cranial surgery.^2^, ^4^ Reported nonseizure advantages of VNS include improved quality of life, alertness, verbal communication, memory, and mood.^6^, ^7^ In the UK, the National Institute for Health and Care Excellence (NICE) recommends VNS as an add‐on treatment to ASM if resective surgery is unsuitable for individuals with DRE^8^; this is irrespective of seizure classification. NICE has also published a review of the evidence underpinning the clinical recommendations for VNS in DRE,^9^ which highlights the lack of robust evidence and the need for further research to assess the effectiveness of VNS in DRE.

It is imperative that we have better individual‐level predictors of VNS success, because unlike failed ASM trials, there is a surgical legacy from VNS failure. Predictive and prognostic models would be helpful for patient selection and stratification, as well as tailored stimulation programming. Presurgical evaluations are commonplace prior to VNS insertion and could be used to retrospectively identify those who might have greater odds of response. Predictive models using such data are widespread both within epilepsy research for predicting outcome from resection^10^, ^11^, ^12^, ^13^, ^14^, ^15^, ^16^, ^17^, ^18^, ^19^, ^20^ and for diagnosis and treatment of neuropsychiatric disorders.^21^, ^22^, ^23^ Here, we discuss potential predictive and prognostic factors for outcome from VNS in adults and children with DRE, which could be used to optimize patient selection for VNS.

## 2 POTENTIAL MARKERS OF VNS RESPONSE

### 2.1 Methodology

We undertook a narrative review to identify consensus points and areas of uncertainty. We used key search terms (Tables S1–S5) and followed with forward and backward citations of the identified studies. The initial draft of identified papers, data sources, and narratives were circulated to all authors to identify potential biases and any key papers that were not detected from the search strategy. We specifically identified data concerning the different modalities, including VNS mechanism of action and neurophysiological, imaging, clinical, and biochemical markers. When sources disagree, we present both narratives. Table 1 and Figure S1 summarize predictive studies with their sample sizes and accuracies.

**TABLE 1:** Summary of identified previous EEG, MEG, and MRI studies predicting response.

Paper	Modality	Sample size and accuracy	Pre‐ or postimplant	Finding
Ma et al. (2022)^39,a^	EEG	70 testing patients 18 validation patients 61.4% w/o clinical features 75.7% w/ clinical features 61.1% on validation	Pre	Differences in PLI between responders and non‐responders
Babajani‐Feremi et al. (2018)^40,a^	MEG	23 patients 89 controls 87% predicting for responder, nonresponder, or control	Pre	Differences in graph measures using PLV between controls, responders, and non‐responders
Sangare et al. (2020)^41^	EEG	35 patients NA accuracy	Post	Differences in graph measures using PLV between responders and non‐responders
Okamura et al. (2020)^43^	MEG	15 patients 0.898 AUC 86.7% accuracy	Pre	Higher number of preimplant bilateral spikes associated with positive response
Guo et al. (2023),^44^	EEG	93 patients NA accuracy	Pre	Higher number of IEDs are associated with positive response
Mithani et al. (2020)^53,a^	MEG	36 testing patients 12 validation patients 88.9% accuracy 0.93 AUC 67% accuracy in validation cohort	Pre	Differences in right‐side SEF activation and functional connectivity between responders and non‐responders
Hödl et al. (2020)^30^	EEG	13 patients NA accuracy	Upon implant	Differences in p3b component between responders and non‐responders
Wostyn et al. (2017)^35^	EEG	18 patients NA accuracy	Post	Differences in P3 amplitude when VNS system was turned on
De Taeye et al. (2014)^29^	EEG	20 patients NA accuracy	Post	P3 peak amplitude changes in responders when VNS system turned on but constant in non‐responders
Arya et al. (2014)^54^	MRI	43 patients 81.2% accuracy lesional 78.3% accuracy nonlesional	Pre	Presence of brain lesion increases likelihood of response
Ghaemi et al. (2010),^56^ Hava Ozlem et al. (2017)^55^	MRI	144, 17 patients NA accuracy	Pre	Presence of brain lesion does not increase likelihood of response
Ghaemi et al. (2010)^56^	MRI	144 patients NA accuracy	Pre	Cortical dysgenesis significantly more common in patients who become seizure‐free
Xie et al. (2023)^57^	MRI	95, 84, 62 patients at 12, 18, 24 months of follow‐up NA accuracy	Pre	No significant difference in distribution of responders and non‐responders for any specific structural etiology
Guo et al. (2023),^44^	MRI	93 patients NA accuracy	Pre	Unilateral encephalomalacia as opposed to bilateral encephalomalacia in responders
Guo et al. (2023)^58,a^	T1w MRI	44 VNS patients 45 drug‐treated patients NA accuracy	Pre	Higher seizure frequency preimplant in non‐responders; structural etiology more common in responders; correlation between brain‐clinical signature (sourced from brain abnormality) predicted seizure reduction and actual seizure reduction
Mithani et al. (2019)^59,a^	DWI	56 patients 89.5% accuracy .93 AUC	Pre	Higher FA in responders for 11 left‐lateralized white matter tracts
Ibrahim et al. (2017)^36,a^	fMRI	29 training patients 8 validation patients 86% accuracy on training 88% accuracy on validation	Pre	Differences in functional connectivity between the thalami, left insula, and ACC between responders and non‐responders
Zhu et al. (2020)^63,a^	fMRI	14 patients NA accuracy	At implantation and post	Differences in change of left hippocampal connection to the thalami between responders and non‐responders after insertion
Brázdil et al. (2019),^46^ Koritakova et al. (2021),^47^	EEG	60 w/ 22 validation, 8 patients 86.7% accuracy on testing 86.4% accuracy on validation 75% accuracy on cohort of 8 using different device	Pre	Difference in relative band power between responders and non‐responders
Ilyas et al. (2018),^49^ Qin et al. (2024)^48^	Stereo‐EEG	1 patient, 12 patients NA accuracy	Post	Difference in band powers in VNS off and on

Abbreviations: ACC, anterior cingulate cortex; AUC, area under the curve; DWI, diffusion‐weighted imaging; EEG, electroencephalography; FA, fractional anisotropy; fMRI, functional MRI; IED, interictal epileptiform discharge; MEG, magnetoencephalography; MRI, magnetic resonance imaging; NA, not available; P3, P300; PLI, phase lock index; PLV, phase lock value; SEF, somatosensory evoked field; T1w, T1‐weighted; VNS, vagus nerve stimulation. ^a^Study was found using the search criteria in Tables S1–S5.

### 2.2 Neurobiological mechanisms of VNS

The antiseizure activity of VNS is thought to be mediated by modulation of key cranial nuclei via afferent projections of the vagus nerve.

**FIGURE 1:**
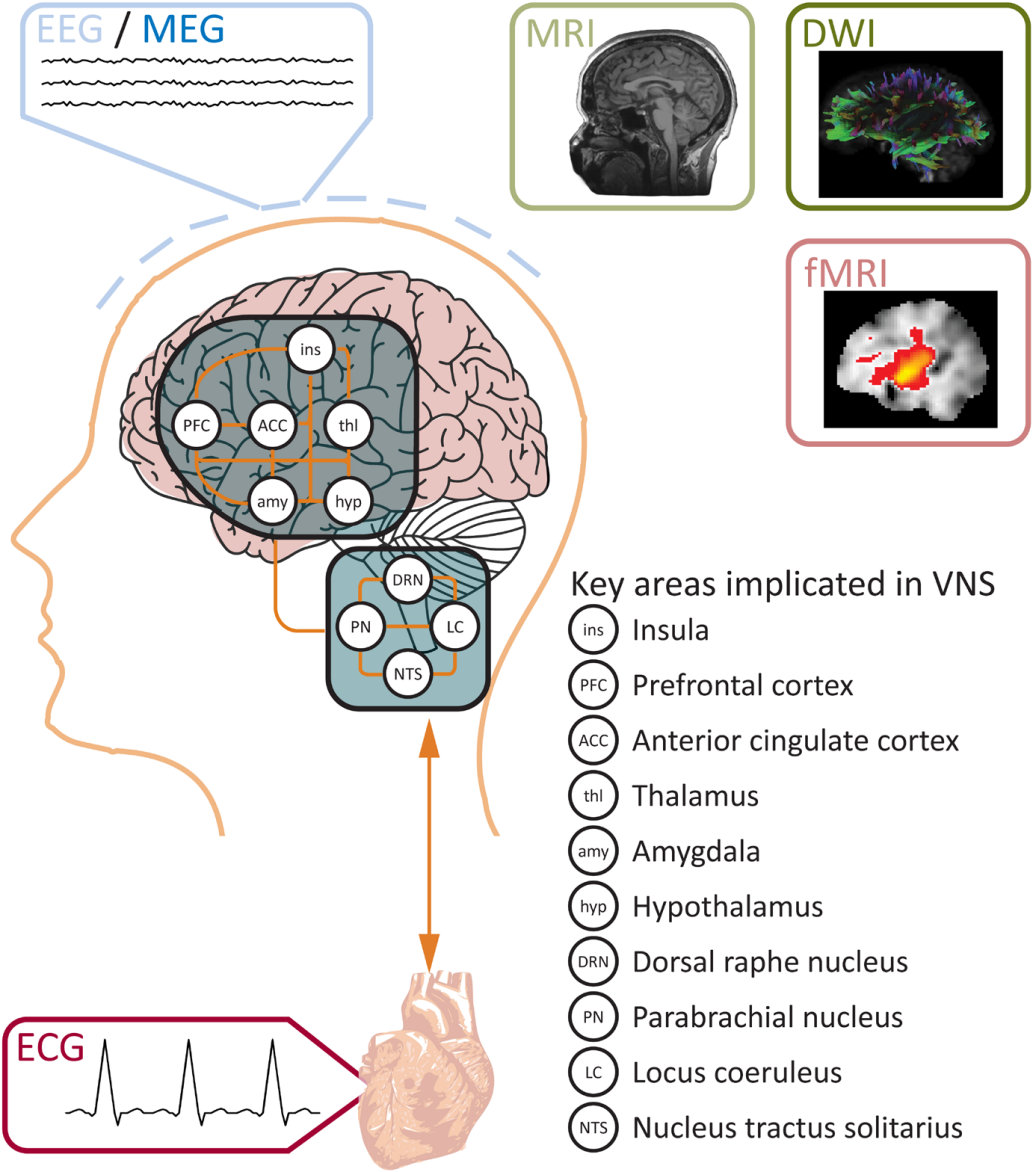
Differences in brain areas found using a variety of modalities point to the mechanistic reason for vagus nerve stimulation (VNS) response. Key areas indicated in response have been discussed within the literature. Abnormality between responders and non‐responders found in any of these areas could be key in identifying both the mechanism of VNS and differences between responders and non‐responders prospectively. This can potentially be done through diverse modalities, although each will relate to distinct underlying biological differences. DWI, diffusion‐weighted imaging; ECG, electrocardiography; EEG, electroencephalography; fMRI, functional MRI; MEG, magnetoencephalography; MRI, magnetic resonance imaging.

The specific brain regions affected by VNS either receive direct vagal nerve afferents or are connected to regions receiving direct afferents (Figure 1). The nucleus tractus solitarius (NTS) is directly stimulated by the vagus nerve,^24^, ^25^ making the NTS a prime candidate for the source of any VNS effects. The NTS projects directly to other brainstem nuclei, specifically the LC and parabrachial nucleus (PN),^26^ which have also been related to the mechanism of action of VNS. The NTS connects to the hypothalamus^27^ and to thalamic and amygdala nuclei.^28^ The LC is connected to the dorsal raphe nucleus (DRN), from where noradrenergic and serotonergic neurons project, with a large impact on widespread cerebral networks^26^ and postulated to be key to VNS response.^29^, ^30^ The PN is closely connected to the NTS, LC, and DRN^31^ and has long‐ranging projections to the hypothalamus, thalamus, and amygdala.^26^ Neuronal activity in the hypothalamus, thalamus, and amygdala have been shown to be affected by VNS^26^, ^28^, ^32^, ^33^, ^34^ and thought to be involved in the mechanism of VNS action.^29^, ^30^, ^35^, ^36^ These regions also connect to the anterior cingulate cortex (ACC), prefrontal cortex, and insula, again implicated in VNS response.^36^ Given their relationship to VNS afferent stimulation and known functional activity, these brain regions are of particular interest for the investigation of VNS prognostic markers.

### 2.3 Neurophysiological markers (electroencephalography, magnetoencephalography)

#### 2.3.1 Functional connectivity

Functional connectivity refers to the statistical relations of brain activity between regions; this measures how synchronized brain activity is when the brain is either at rest or engaged in a task. Functional connectivity measures include the phase lock value (PLV; the absolute difference between the mean phase of two signals) and phase lock index (PLI; the synchronization of phase between two signals). Studies using PLV and PLI, and network metrics derived from them, have seen success in both prognostic indication of VNS response^37^, ^38^, ^39^, ^40^ and postinsertion variance between responders and non‐responders.^41^, ^42^

Preimplantation PLI in the high beta frequency bands range from scalp electroencephalography (EEG) are significantly greater in responders (.165 ± .027) when compared to non‐responders (.152 ± .007).^39^ There are also nonsignificant trends of reductions in PLV, PLI, and weighted PLI for different frequency bands. A machine learning method trained on these synchronization features in 70 patients achieved adequate accuracy (61.4%), with improvements to the accuracy (75.7%) when provided with clinical features. The accuracy was substantially less (61.1%) in a small validation cohort of 18 patients. Therefore, preimplantation PLI from scalp EEG, which is easy to obtain, shows promise but now needs to be evaluated as a marker in larger cohorts, both for training of the model and for prospective validation. Postimplantation,^41^ there were significant differences in PLI and PLV for responders when comparing VNS on and off periods. In non‐responders, there was no significant difference. This further supports exploring PLV and PLI as relevant metrics in VNS.

Graph‐based network measures from magnetoencephalography (MEG) have also been used to identify factors predictive of VNS response. Specifically, resting state MEG graph measures, derived from PLV, varied between controls, responders, and non‐responders.^40^ Responders had significantly greater modularity and lower transitivity when compared to non‐responders across all three tested frequency bands (theta, 4–8 Hz; alpha, 8–12 Hz; beta, 12–30 Hz). These graph measures also placed responders between non‐responders and controls. A machine learning method given the transitivity and modularity, with all three frequency bands as input, achieved promising accuracy (87%) predicting between controls, responders, and non‐responders. The incorrect predictions were primarily for controls and non‐responders being predicted as responders.

Taken together, these results suggest that network neurophysiological measures have the potential to distinguish VNS responders from non‐responders using preimplantation data, and the same effect persists postimplant. At a minimum, this may help identify the two most important ends of the spectrum, differentiating between "superresponders" and people who have little or no response.

#### 2.3.2 Epileptiform discharges

Individuals with more preimplantation bilateral spikes seen on MEG were more likely to be responders to VNS.^43^ Postimplantation, the number of bilateral spikes was significantly reduced in responders, from a median of 22 to 5 (*p* = .03). Conversely, non‐responders did not show a significant change in bilateral spikes from preimplantation levels. The relative reduction in bilateral spikes postimplantation suggests that VNS may reduce the bilateral spread of epileptic activity.

However, there is also evidence that individuals with unilateral instead of bilateral interictal epileptiform discharges seen on EEG are more likely to respond to VNS.^44^ In another study, the absence of bilateral interictal epileptiform discharges was associated with response to VNS.^45^ Patient selection may have biased results, as the Okamura study^43^ contained many patients with corpus callosotomies, the Guo study^44^ exclusively included patients with encephalomalacia, and neither was controlled.

#### 2.3.3 Relative band power

EEG power may help to predict VNS response. Relative mean power differences in the alpha and gamma bands from interictal scalp resting state EEG have been shown to be significantly different between responders and non‐responders.^46^ These differences were used to train a predictive model for the train/test cohort of 60 patients, then validated on 22 patients, which achieved a promising accuracy (86.4%). The model has since been further validated using a different EEG dataset, collected using a different device, in eight new patients.^47^ Stereo‐EEG has also shown differences in VNS on and off periods for individuals with a VNS device already present, although this has not been explored prospectively.^48^, ^49^

Brain symmetry as defined by the pairwise‐derived Brain Symmetry Index (pdBSI)^50^ may differentiate responders from non‐responders to VNS using interictal EEG. pdBSI values were lower for delta, theta, and alpha frequency bands in VNS responders when compared to non‐responders (*p* = .07, .06, and .02, respectively).^51^ However, other studies did not replicate this finding.^52^

#### 2.3.4 Event‐related potentials

Event‐related potentials (ERPs) are electrical brain responses that are time‐locked to specific sensory, cognitive, or motor events. ERPs from decision‐making tasks have been hypothesized to be indicative of VNS response. The amplitude of the P300 (P3) wave was significantly lower for VNS responders than non‐responders with VNS off, but was not significantly different with VNS on.^30^ Increased P3 amplitude was also found in responders when VNS was switched on, whereas non‐responders showed a decrease.^35^ This has been corroborated, as the peak amplitude of P3 changed when VNS was switched on in responders, but was constant in non‐responders.^29^ These findings suggest that postimplant ERPs differentiate response groups. P3 has not been studied preimplantation as a prognostic indicator of response, to our knowledge.

The median nerve has substantial overlap with the circuitry of the afferent vagus nerve. Because the median nerve is arguably more easily accessible peripherally, this has enabled the experimental use of median nerve stimulation as a proxy for the VNS in MEG studies. Median nerve stimulation produces somatosensory evoked fields (SEFs) within the somatosensory cortex, which can be localized with MEG.^53^ The distance between the right somatosensory cortex and maximal right‐side activation was significantly different between responders and non‐responders to VNS (*p* = 3 × 10^−4^), although the same effect was not seen on the left.^53^ The same study^53^ also reported greater connectivity and event‐related peak for the beta and gamma band in responders compared to non‐responders. With right median stimulation, responders when compared to non‐responders showed greater connectivity in the frontal and limbic circuitry in the beta band. With left median stimulation, responders also showed greater connectivity in the sensorimotor network in the gamma band. A machine learning method trained on the SEF characteristics and functional connectivity obtained high accuracy (88.9%) but did not translate as well to a validation cohort of 12 (67%). These results show potential for the use of median nerve stimulation as a noninvasive proxy during evaluation of patients for VNS.

### 2.4 Imaging markers

#### 2.4.1 Structural abnormalities

The evidence on the predictive value of magnetic resonance imaging (MRI)‐visible abnormality remains contentious. Initial reports stated that structural brain lesions predict a less favorable VNS response^54^; however, this finding was later refuted.^55^, ^56^ Cortical dysgenesis on MRI was significantly more common in those who were seizure‐free after VNS (*p* = .004).^56^ Another study described that unilateral when compared to bilateral encephalomalacia was predictive of VNS response.^44^ However, on yet further study, no differences were found in the distribution of VNS responders and non‐responders with any specific structural lesion.^57^

Due to the palliative use of VNS, these studies used data from patients with structural pathology that was deemed not amenable to resection. There is a lack of data allowing the comparison of VNS cohorts to wider DRE groups. We conclude that selection bias may therefore significantly affect these results. The influence of specific structural brain lesions on VNS response remains unclear and needs a definitive answer, at either a group or subgroup level.

#### 2.4.2 Structural networks

Network characteristics from structural abnormality may predict response to VNS. Several brain network properties can be calculated, including small‐worldness and global efficiency.^58^ Network properties were computed from a weighted matrix of the deformation, in 22 responders and 23 VNS non‐responders, combined with clinical variables and referred to as “the brain‐clinical signature,” which was then used in several predictive and associative models.^58^ In one model, percentage seizure reduction was predicted from the brain‐clinical signature (*R* = .58, *p* < .01). Another model found that a higher seizure frequency preimplant increased the likelihood of nonresponse. It was also found that a structural abnormality as the primary cause of DRE, rather than genetic, metabolic, infectious, or unknown causes, was associated with better VNS response,^58^ although no specific structural causes were investigated.

#### 2.4.3 Fractional anisotropy (diffusion‐weighted imaging)

Fractional anisotropy (FA) is used to quantify the integrity of white matter tracts in the brain. In a sample of 38 children, greater FA was noted in 11 distinct white matter structures in the left hemisphere of responders when compared to non‐responders.^59^ These FA values place responders closer to known control values.^60^, ^61^, ^62^ A machine learning method provided promising accuracy (83.3%) on a validation cohort of 18 patients. Caution should be taken due to the limited sample size. Overall, this suggests that greater deviation from healthy control values is associated with worse VNS response, consistent with EEG findings.

#### 2.4.4 Functional connectivity (functional MRI)

Functional connectivity derived from functional MRI (fMRI) has also been investigated as a marker of VNS response. VNS responders, when compared to non‐responders, have been described as having significantly greater functional connectivity between the thalami, left insula, and ACC.^36^ These differences in functional connectivity were used to train a machine learning model, which obtained high accuracy on the training cohort of 21 (86%) as well as on an external validation cohort of eight (88%). However, the validation cohort only contained one poor VNS responder, which was predicted incorrectly as a responder. The usefulness of fMRI in presurgical evaluation for VNS has been further supported by the finding of lower left hippocampal–thalamic connections for seven responders when compared to seven non‐responders.^63^

### 2.5 Clinical and biochemical markers

#### 2.5.1 Seizure history and timing of VNS implantation

VNS outcomes based on age at seizure onset and at VNS implantation have been studied. In 45 children, the implantation age (dichotomized at 5 years) had no effect on postimplantation seizure frequency or ASM use; however, the younger patient group had better cognitive and quality of life outcomes.^64^ In both pediatric and adult populations, shorter durations of epilepsy preimplant have been associated with improved response rates to VNS.^3^, ^54^, ^65^, ^66^ Those with a duration of epilepsy diagnosis of <12.5 years prior to VNS implantation, compared to those with longer duration of disease, had significantly higher VNS response rates.^67^ This positive differential effect on VNS response appears to be lost when the duration of epilepsy preimplantation is >15 years.^44^ In contrast, it has been reported, both in a VNS registry^68^ and in a separate cohort of 158 patients,^69^ that older age and longer duration of epilepsy were associated with better outcomes. Interpretation of these data should be cautious due to the inevitable effects of selection bias in uncontrolled studies. We speculate that a shorter duration of uncontrolled epilepsy before VNS implantation allows for less time for an abnormal circuitry to be established.

One study reported a higher preimplant seizure frequency correlated with improved response to VNS.^70^ However, without a control group, this may be the reflection of a temporary increase in seizures, with the natural history for them to settle down irrespective of the use of VNS.^70^ Furthermore, other studies provide contradictory findings.^58^, ^68^, ^69^ For example, reported preimplantation seizure frequency was negatively associated with VNS response in 44 patients.^58^ Longer term prospective and controlled studies of the natural history of DRE, and the impact of VNS, are required to elucidate this relationship.

A history of resective surgery does not appear to be a poor prognostic indicator for VNS^71^; 50% of postsurgical patients, compared to 67% of patients with no prior surgery, responded to VNS.^72^, ^73^ This suggests that VNS should be considered in patients who have not had effective seizure control following surgical resections, or conversely that surgical resection is not a relative contraindication for VNS.

#### 2.5.2 Physiological markers of vagal function

Although the mechanism of action of VNS is not fully understood, markers of vagal function, such as proxy measures of vagal tone, may relate to therapeutic response.

Heart rate variability (HRV) is a physiological marker of autonomic function. Given the role of the vagus nerve in regulating autonomic function, and the relative simplicity of computing HRV metrics from electrocardiograms (ECGs), this is a commonly used metric. There are multiple measures of HRV relating to the interval between successive heartbeats (RR intervals) and derivatives thereof, primarily divided into time and frequency domains.^74^ Altered HRV is known to be associated with DRE,^75^ but its association with outcome from VNS is variable. DRE patients, compared to healthy controls, have been described as having significantly lower HRV complexity indices, as well as traditional linear HRV measurements,^76^, ^77^ agreeing with previous evidence.^75^ The preoperative root mean square of differences in successive RR intervals, a commonly used time domain HRV metric, appears to have the greatest discriminatory power in determining VNS responders and non‐responders.^76^ Additionally, linear HRV parameters were significantly lower than in controls, although this did not correlate with VNS outcome.^76^ Multiscale entropy appears to be increased in VNS responders, but this could be an effect of VNS rather than an underlying predictor of response.^77^

VNS responders, when compared to non‐responders, have been described to have significantly lower HRV high‐frequency power, both at baseline and after 1 year of VNS.^30^ This suggests that the high‐frequency power measurement of HRV is associated with responsiveness to therapy and could be mechanistically related to the effects of VNS that led to seizure reduction. For patients who did not respond to VNS at 12 months, there was a decrease in the SD of RR intervals, SD of successive RR difference, and SD of the nonlinear variables in the preictal phase, which was not seen in patients who responded to VNS.^78^ This decrease may reflect a preictal autonomic imbalance, which VNS does not influence.

A machine learning study of HRV data suggested that 35 of the 59 tested ECG features measured during sleep were significantly associated with response to VNS.^79^ The authors hypothesize that during sleep the parasympathetic vagal markers may be more prominent than when awake, which may relate to autonomic circadian rhythm. Extended recording of ECG over longer periods of time and during sleep may be more valuable in predicting response to VNS. A further complexity in analysis arises here from the need to differentiate direct efferent effects of VNS on heart rate (which seem unlikely to relate to seizure control) and indirect effects on HRV from central autonomic modulation produced by afferent effects of VNS. Current analysis algorithms cannot control for this. Generally, improved HRV metrics with treatment may be associated with better VNS outcomes, but the evidence is mixed and at times contradictory.^80^

#### 2.5.3 Biochemical markers of vagal function

Vagal stimulation has been hypothesized to modulate the immune system via many different mechanisms, including decreasing neurotoxic cytokine levels and increasing levels of tryptophan. Plausibly, this may be as a primary or more likely as a secondary mechanism. Several studies have investigated these markers in peripheral blood and cerebrospinal fluid (CSF) samples, comparing responders with non‐responders to VNS, without consistent results.

In peripheral blood, VNS responders had a decrease in proinflammatory cytokines postimplantation compared to preimplantation.^32^ This is correlative and does not establish causation. Cytokine levels in the blood and CSF samples were comparable at baseline in a group of patients randomized to high or low VNS stimulation.^32^ Lower baseline plasma IL‐6 levels were associated with a greater reduction in seizure frequency and preimplantation CSF levels; however, they were not correlated with reduction in seizure frequency.^81^ After VNS treatment, high levels of anthranilic acid, a neuroprotective and anticonvulsant tryptophan metabolite, were associated with increased percentage seizure reduction,^82^ in contrast to a study in which a correlation was not found.^32^ In conclusion, the role of the inflammatory and biochemical pathways in VNS response is interesting but has not been established.

#### 2.5.4 Stimulator settings

Underdosing of VNS may explain some of the lack of response described in observational clinical cohorts, and it is possible that there is scope for further uptitration and optimization of stimulator settings to improve VNS outcomes.^83^ Animal studies support trialing different VNS stimulation paradigms to increase efficacy of seizure control. Rat studies compared hippocampal field activity in response to rapid, and standard cycle, VNS stimulation. With rapid cycles, there was greater reduction in hippocampal EEG power, therefore desynchronization, and this may be related with better seizure control.^84^ Studies in rats recording LC activity in response to different VNS settings found differential activation according to the parameters of simulation delivered.^85^ Studies in animals have enabled invasive monitoring to be undertaken alongside stimulation, allowing for the study of the induced electrical changes. These studies also add weight to the hypotheses of mechanism of action of VNS, by demonstrating in vivo changes elicited in various brain areas, related to VNS. This provides further evidence for using different stimulation paradigms, but there will be challenges to translation into humans.

Human studies suggest that VNS settings and titration protocols could be optimized.^86^ Rapid duty cycling (off time ≤ 1.1 minute, while keeping duty cycle < 50%) was associated with greater efficacy, with the response rate (>50% reduction in seizures) increasing from 45% to 77%.^87^ Response in adult and pediatric cohorts to different stimulation paradigms may differ and should be noted when programming VNS. A study comparing rates of titration to an empirically allocated 1.625‐mA output current, delivered with pulse widths of 250 μs and frequencies of 20 Hz (all reasonably within “standard” titration target), was conducted in adults and children. Speed of titration was grouped into three cohorts, with duration to maximal therapy being <3 months, 3–6 months, or >6 months. In children, a more rapid titration of VNS therapy was tolerated and led to a faster improvement in seizures, whereas in adults increased speed of titration was not well tolerated. Patient age and duration of epilepsy before implantation were not found to affect response to rate of titration.^88^ These studies provide insight in different patient groups for the effects of diverse titration paradigms and can guide clinical programming to optimize seizure control and time to VNS effect onset. However, in clinical practice guidance is essentially homogeneous, with deviation being guided by clinical acumen, with little evidence to guide choices.

VNS devices with AutoStim, in which the generator can be automatically triggered to start an on period outside its background duty cycle, in response to preset heart rate increase thresholds, are leading to greater rates of seizure reduction.^89^ Specifically, this is done through cardioresponse, with ictal tachycardia activity thresholds that are individually optimized.^90^, ^91^, ^92^ This was initially supported by the benefit seen from manual magnet stimulation, whereby a magnet is used to turn the VNS device on outside of its background duty cycle, in response to the patient noticing seizure activity.^93^ In theory, this implies the ability of a VNS stimulator to not only prevent but also to stop active seizures.

## 3 DISCUSSION

There are heterogeneous studies and at times contradictory findings relating to the predictive factors for VNS response. Significant findings for an increased likelihood of response are summarized in Figure S1.

It is important to identify reliable predictive markers for VNS response, as currently none is in clinical use. A natural starting point is the use of clinical features and data from EEG and T1‐weighted MRI, which are routinely acquired in people with epilepsy. Resting state fMRI and fluorodeoxyglucose positron emission tomography will also be available in many subjects.

**FIGURE 2:**
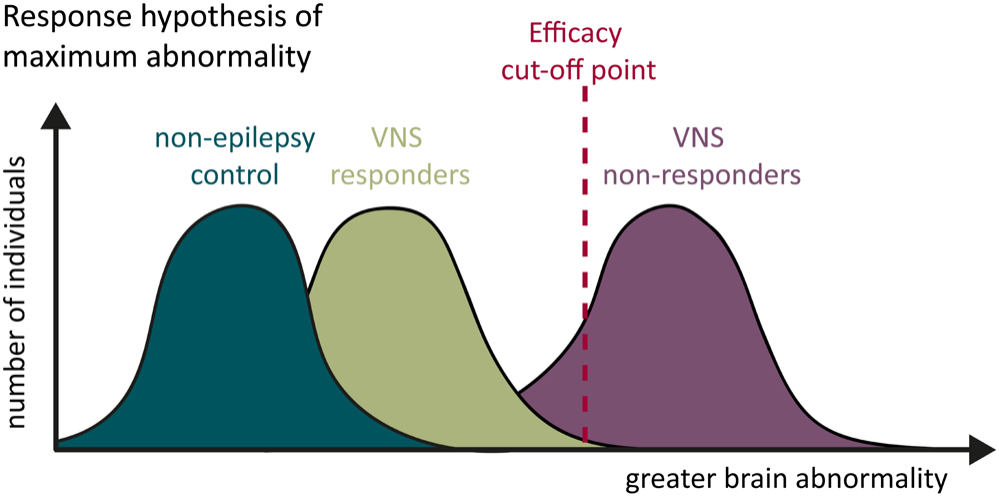
When is vagus nerve stimulation (VNS) effective? VNS effectiveness may be due to the varied abnormality between responders and non‐responders. Non‐responders show greater abnormality than responders across a variety of metrics.^40^, ^59^ We hypothesize that there is point of maximum abnormality where individuals with epilepsy will no longer respond to VNS therapy.

Some sources corroborate the concept that on preimplantation measures, responders were more similar to healthy controls than non‐responders.^40^, ^59^ This suggests that focusing on markers of poor response rather than those of good response may provide better results. This supports the response hypotheses of maximum abnormality (Figure 2). This is not the case for all biomarkers, as other studies show responders as being further from healthy controls such as with high‐frequency HRV.^30^ Resemblance of responders to control cohorts is still uncertain but appears to be a promising avenue for further research.

### 3.1 Limitations

There is little consensus in the literature surrounding predictors of VNS response. Studies have often been small, originating from single centers and not controlled.

Many studies compare VNS responders and non‐responders, which is useful to determine prognostic factors. Some studies reviewed did not compare responder groups but considered various parameters such as stimulation paradigms. These are not applicable for prognostication but have value in understanding the mechanism of action of VNS and improve outcomes. Furthermore, improved mechanistic understanding could be helpful in device development, such as transcutaneous and other noninvasive VNS techniques.^94^, ^95^, ^96^, ^97^ The availability of noninvasive VNS techniques also opens the door to preclinical studies in humans focusing on mechanistic exploration.

Another confound is that stimulation parameters were not regularly collated. Clinical devices allow for individual configuration of current output, pulse widths, frequency of stimulation, and duty cycles. Newer devices further allow for different settings during nighttime. The setting space is therefore wide and complex to study systematically. Underdosing of VNS may explain differential outcomes. Further prospective datasets collecting stimulator settings would help clarify optimum stimulation settings.

A responder is usually defined as >50% reduction in seizure frequency; however, seizure reduction only tells part of the story. Reduced seizure severity, need for hospitalizations, dosages, number of ASMs, sudden unexpected death in epilepsy risk, and improved mood are all important.^98^, ^99^, ^100^, ^101^, ^102^ The effects of VNS on mood might be particularly relevant in this population, given the high rates of comorbidity of epilepsy and major depressive disorder, and its effects on quality of life.^103^ It is of note that VNS is also used as an augmentation therapy in the management of treatment‐resistant depression in people without epilepsy.^104^ A more suitable definition of a desirable outcome from VNS may therefore be broader than seizure reduction, and measuring the effect of various prognostic markers using a more holistic measure would be valuable, with a focus on overall quality of life. Considering brain–body interoception may be useful in this context.^105^ The focus of this narrative review is on VNS efficacy for seizure reduction, rather than tolerability and short‐ or long‐term side effects. However, holistically, it is worth considering that VNS lacks somatic side effects such as contributing to metabolic syndrome, changes in bone mineralization, or even teratogenic effects.^106^ Additionally, response is currently defined at one singular time point usually between 1 and 5 years postimplantation. It is known that response rates improve over time,^4^ but loss of response can also occur. Studies looking into the reasoning for both this increase in response and loss of response would be useful in understanding the mechanism of VNS clinically.

There are inherent problems when assessing efficacy due to the inaccuracy of seizure self‐reporting.^107^ In a systematic review of the effects of placebo compared to ASM, placebo was associated with 50% reduction in seizures in 15% of patients.^108^ In the context of VNS, this would represent approximately one third of responders. Better methods to capture seizure occurrence more accurately, such as using wearable devices, may alleviate this issue. It is also worth noting that high rates of placebo response, as well as challenges in blinding, are common in medical device trials.

There is significant selection bias in patients who are offered VNS clinically, many of whom have an intellectual disability and who do not necessarily represent the same cohort as those assessed for definitive resective surgery. This should be considered when extrapolating results to a wider cohort of patients with DRE. Patients in whom resection is not appropriate due to inability to define a resectable epileptogenic zone, or because the region is not accessible without a high risk of causing deficit, are the primary candidates for VNS implantation. Predictive models will need to account for the heterogeneity of cohorts and be trained on more diverse cohorts or be designed to have an awareness of these features.

### 3.2 Potential applications and future work

**FIGURE 3:**
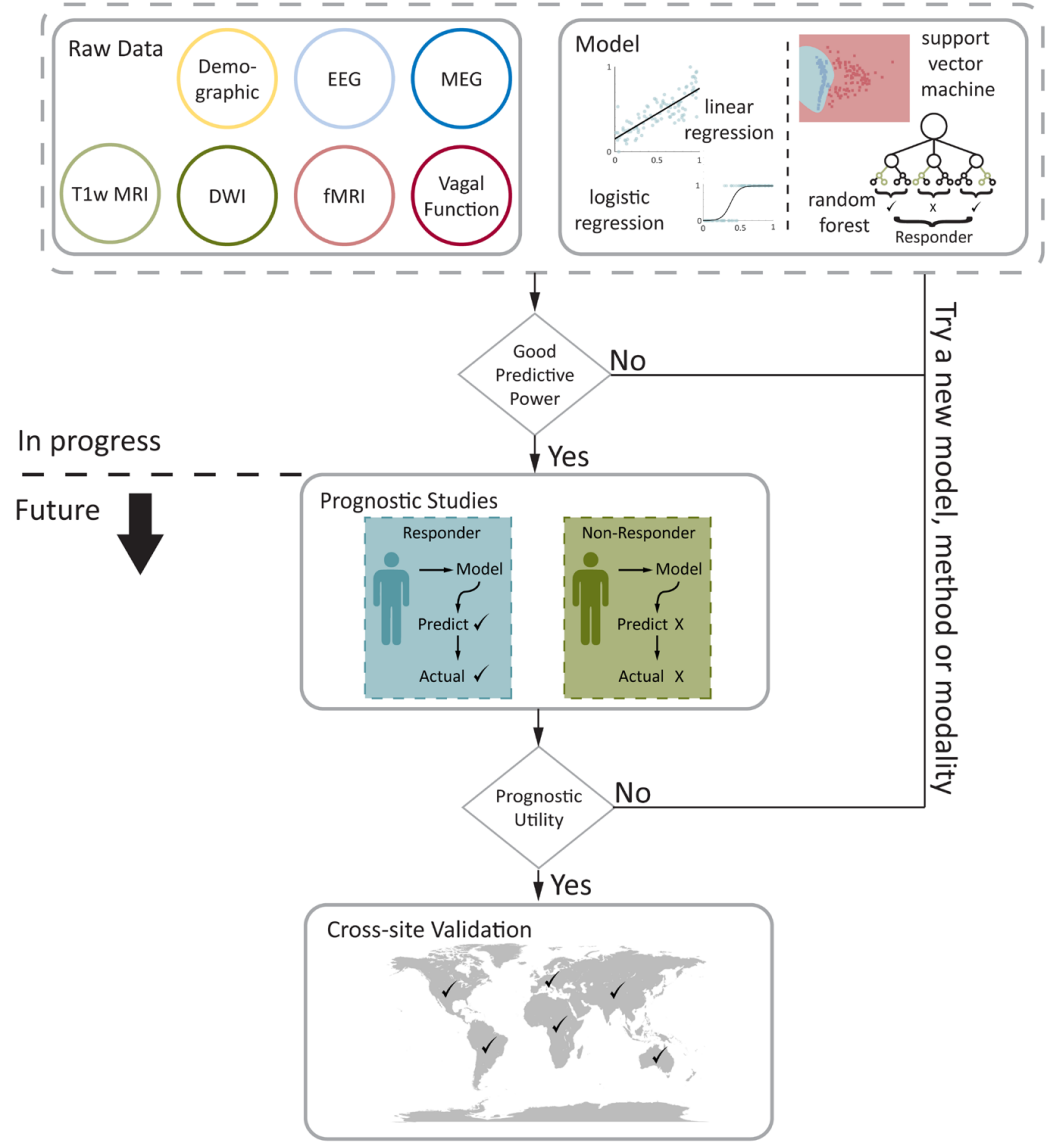
Translating predictive models into clinical practice. A number of promising models from a range of different data have been described. However, for these markers to be used in clinical practice, their predictive power needs to be demonstrated prospectively and across sites globally. Work is still required to determine the most effective markers for each modality and the optimum use in combination to predict patient outcome. DWI, diffusion‐weighted imaging; EEG, electroencephalography; fMRI, functional MRI; MEG, magnetoencephalography; MRI, magnetic resonance imaging; T1w, T1‐weighted.

For the development of accurate and clinically applicable predictive models, findings need to be replicated prospectively in larger cohorts and controlled trials, preferably covering a range of centers, scanners, and protocols, as well as patient cohorts, to provide robustness to the findings (Figure 3). Large datasets are also valuable, such as the CORE‐VNS registry^109^ and CONNECTiVOS study.^110^ The CONNECTiVOS study aims to gather MRI, diffusion‐weighted imaging (DWI), and fMRI as well as MEG and SEFs at centers with MEG scanners to verify previous findings.^53^, ^110^ The aim is to use these data to identify markers of response. We are not aware of an equivalent study in adults, although there is a planned prospective cohort.^111^ Focusing on real‐world clinical data, VNS for major depressive disorder has an ongoing unified European prospective registry (RESTORE‐LIFE), collecting standardized baseline and outcome data from 15 centers over 5 years postimplantation, offering a model that could be easily translated to the epilepsy space.^112^

Machine learning methods can assist in generating decisions from data when standard delineation may not be possible. This could lead to more precise prediction of outcomes. Published methods in this space have been limited to general statistical testing and the training of linear classifiers such as support vector machines. Using deep learning methods such as convolutional neural networks or graph neural networks, better prediction may be possible. These methods, however, may make it more difficult to interpret why or how certain predictions are made.

An area of active research for epilepsy is the use of virtual brain modeling. This approach uses sets of equations, capable of replicating seizurelike dynamics, which are constrained by parameters.^113^, ^114^, ^115^ The parameters are typically taken from the individual patient's EEG or MRI data, and thus create a digital twin in which treatments such as surgery can be simulated.^116^, ^117^, ^118^, ^119^ Although some studies have investigated the effect of stimulation in virtual brain models,^120^, ^121^ only one explicitly modeled the impact of vagal stimulation on seizure dynamics.^122^ We expect future personalized models and stimulation protocols for improved prediction.

## 4 CONCLUSIONS

Many biomarkers of VNS response are actively being studied, but currently, no individual or group of markers can be used prospectively to provide accurate prediction of response. Given the heterogeneity of epilepsy and variability of seizures even within an individual, we anticipate multiple markers may be needed, which may reflect different mechanisms of action in different patients. Promising findings are, however, present across electrical, imaging, and physiological modalities, including EEG, MEG, MRI, DWI, fMRI, and vagal function, such as HRV. Although demographic factors appear to have poor predictive power, they may be useful as adjuncts to other modalities to delineate groups most likely to respond to VNS. With improved predictive models based on personalized data, we envisage the scenario of VNS use as an adjunct therapy to reduce ASM load and minimize consequential side effects.^123^ Future research is likely to produce better individual‐level predictors of VNS success.

## AUTHOR CONTRIBUTIONS

**All authors:** Substantial contributions to the conception or design of the work and interpretation of data for the work. **All authors:** Drafting the work or revising it critically for important intellectual content. **All authors:** Final approval of the version to be published. **All authors:** Agreement to be accountable for all aspects of the work in ensuring that questions related to the accuracy or integrity of any part of the work are appropriately investigated and resolved.

## CONFLICT OF INTEREST STATEMENT

None of the authors has any conflict of interest to disclose. We confirm that we have read the Journal's position on issues involved in ethical publication and affirm that this report is consistent with those guidelines.

## Supporting information


appendix S1.

## Data Availability

Data sharing not applicable to this article as no datasets were generated or analysed during the current study.
