# Supplementary material for: Vagus nerve stimulation for epilepsy: A narrative review of factors predictive of response
Source: Epilepsia. 2024 Oct 16;65(12):3441–56. doi: 10.1111/epi.18153 (PMC11647441; doi:10.1111/epi.18153)
Supplement: Supplementary file 1 — appendix S1. [file EPI-65-3441-s001.docx]

Supplementary Material

# Search and study inclusion criteria

As with this review the goal of the search was to find papers predicting response to VNS using data acquired prior to surgical implantation. Therefore, the search used aimed to find papers which fit the following criteria:

1. A study of Epilepsy patients
2. A study of patients implanted with VNS devices
3. A study using the specified modality (EEG, MEG, MRI, DWI, or fMRI)
4. A study that finds a marker predictive of response

To fulfil these criteria the following search items were used:

1. Epilepsy OR DRE
2. VNS OR Vagus Nerve Stimulation OR Vagus Nerve Stimulator
3. All names for appropriate modality (shown in text above tables 2-6)
4. Predict OR predictor OR predictive OR prediction OR marker OR biomarker

This was done both by systematic search (as described above) and through hand searches of reference lists from relevant articles. Details on papers found in each modalities individual search can be found in supplementary tables 2-6. Due to the wide variety of options within papers on vagal function and demographics similar searches weren’t performed for these modalities and instead were found through non-systematic searching and hand searches of references lists.

# Supplementary Figure 1 predictive power across modalities:

**
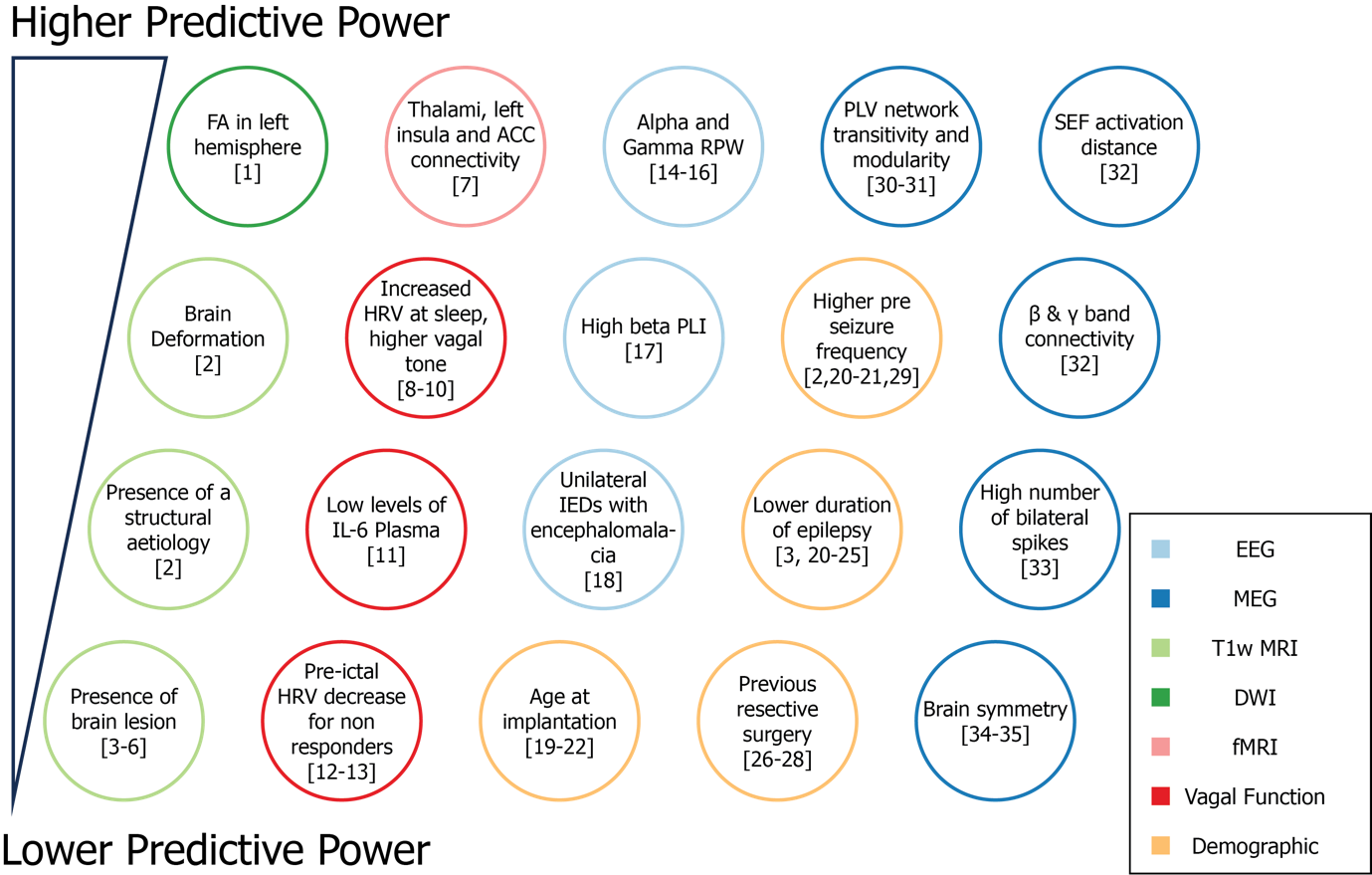
**

**Markers of VNS response are within a variety of modalities with varied predictive power**. Modalities show differing predictive power depending on the measure used. Specifically demographic factors have relatively low predictive power in isolation so are only useful for prediction in combination with other factors.

Abbreviations: ACC Anterior Cingulate Cortex, DWI Diffusion Weighted Imaging, EEG Electroencephalography, FA Fractional Anisotropy, fMRI Functional Magnetic Resonance Imaging, HRV Heart Rate Variability, IED Interictal Epileptiform Discharges, MEG Magnetoencephalography, MRI Magnetic Resonance Imaging, P3 p300, PLI Phase Lag Index, PLV Phase Lag Value, RPW Relative Power Weight, SEF Somatosensory Evoked Field

References:

1. Mithani, K., et al., *Connectomic Profiling Identifies Responders to Vagus Nerve Stimulation.* Annals of Neurology, 2019. **86**(5): p. 743-753.
2. Guo, Z., et al., *Brain‐clinical signatures for vagus nerve stimulation response.* CNS Neuroscience &amp; Therapeutics, 2023. **29**(3): p. 855-865.
3. Arya, R., et al., *Predictors of response to vagus nerve stimulation in childhood-onset medically refractory epilepsy.* Journal of child neurology, 2014. **29**(12): p. 1652-1659.
4. Hava Ozlem, D., et al., *Research Article Vagus Nerve Stimulation in Adults With Drug-Resistant Epilepsy: Efficacy, Adverse Effects and Outcomes.* Journal of Neurological Sciences [Turkish], 2017. **34**(4): p. 60.
5. Xie, H., et al., *Efficacy of vagus nerve stimulation in 95 children of drug-resistant epilepsy with structural etiology.* Epilepsy & Behavior, 2023. **140**: p. 109107.
6. Ghaemi, K., et al., *Vagus nerve stimulation: Outcome and predictors of seizure freedom in long-term follow-up.* Seizure, 2010. **19**(5): p. 264-268.
7. Ibrahim, G.M., et al., *Presurgical thalamocortical connectivity is associated with response to vagus nerve stimulation in children with intractable epilepsy.* NeuroImage: Clinical, 2017. **16**: p. 634-642.
8. Liu, H.-Y., et al., *Preoperative Heart Rate Variability as Predictors of Vagus Nerve Stimulation Outcome in Patients with Drug-resistant Epilepsy.* Scientific Reports, 2018. **8**(1).
9. Liu, H., et al., *Chronic vagus nerve stimulation reverses heart rhythm complexity in patients with drug-resistant epilepsy: An assessment with multiscale entropy analysis.* Epilepsy & Behavior, 2018. **83**: p. 168-174.
10. Madhani, S.I., et al., *Electroencephalogram and heart rate variability features as predictors of responsiveness to vagus nerve stimulation in patients with epilepsy: a systematic review.* Childs Nervous System, 2022. **38**(11): p. 2083-2090.
11. Aalbers, M.W., et al., *The Effects of Vagus Nerve Stimulation on Pro- and Anti-Inflammatory Cytokines in Children with Refractory Epilepsy: An Exploratory Study.* Neuroimmunomodulation, 2012. **19**(6): p. 352-358.
12. Hödl, S., et al., *Pre-ictal heart rate variability alterations in focal onset seizures and response to vagus nerve stimulation.* Seizure, 2021. **86**: p. 175-180.
13. Hödl, S., et al., *Neurophysiological investigations of drug resistant epilepsy patients treated with vagus nerve stimulation to differentiate responders from non‐responders.* European Journal of Neurology, 2020. **27**(7): p. 1178-1189.
14. Brázdil, M., et al., *EEG reactivity predicts individual efficacy of vagal nerve stimulation in intractable epileptics.* Frontiers in Neurology, 2019. **10**: p. 392.
15. Korit́áková, E., et al., *A novel statistical model for predicting the efficacy of vagal nerve stimulation in patients with epilepsy (Pre-X-Stim) is applicable to different EEG systems.* Frontiers in Neuroscience, 2021. **15**: p. 635787.
16. Dolezalova, I., et al., *PRediction of vagal nerve stimulation EfficaCy In drug-reSistant Epilepsy (PRECISE): prospective study for pre-implantation prediction/study design.* Frontiers in Neurology, 2022. **13**: p. 839163.
17. Ma, J., et al., *A prediction model integrating synchronization biomarkers and clinical features to identify responders to vagus nerve stimulation among pediatric patients with drug‐resistant epilepsy.* CNS Neuroscience &amp; Therapeutics, 2022. **28**(11): p. 1838-1848.
18. Guo, M., et al., *Vagus nerve stimulation for pharmacoresistant epilepsy secondary to encephalomalacia: A single-center retrospective study.* Frontiers in Neurology, 2023. **13**: p. 1074997.
19. Soleman, J., et al., *Improved quality of life and cognition after early vagal nerve stimulator implantation in children.* Epilepsy & Behavior, 2018. **88**: p. 139-145.
20. Riestenberg, R.A., et al., *Patient-specific characteristics associated with favorable response to vagus nerve stimulation.* World neurosurgery, 2022. **161**: p. e608-e624.
21. Labar, D., *Vagus nerve stimulation for 1 year in 269 patients on unchanged antiepileptic drugs.* Seizure, 2004. **13**(6): p. 392-398.
22. Englot, D.J., E.F. Chang, and K.I. Auguste, *Efficacy of vagus nerve stimulation for epilepsy by patient age, epilepsy duration, and seizure type.* Neurosurgery Clinics, 2011. **22**(4): p. 443-448.
23. Zhu, J., et al., *Epilepsy duration as an independent predictor of response to vagus nerve stimulation.* Epilepsy Research, 2020. **167**: p. 106432.
24. Wang, H.-j., et al., *Predictors of seizure reduction outcome after vagus nerve stimulation in drug-resistant epilepsy.* Seizure, 2019. **66**: p. 53-60.
25. Russo, A., et al., *Early implantation as a main predictor of response to vagus nerve stimulation in childhood-onset refractory epilepsy.* Journal of Child Neurology, 2021. **36**(5): p. 365-370.
26. Amar, A.P., M.L. Apuzzo, and C.Y. Liu, *Vagus nerve stimulation therapy after failed cranial surgery for intractable epilepsy: results from the vagus nerve stimulation therapy patient outcome registry.* Neurosurgery, 2008. **62**: p. SHC-506-SHC-513.
27. Tecoma, E.S. and V.J. Iragui, *Vagus nerve stimulation use and effect in epilepsy: what have we learned?* Epilepsy & Behavior, 2006. **8**(1): p. 127-136.
28. Elliott, R.E., et al., *Impact of failed intracranial epilepsy surgery on the effectiveness of subsequent vagus nerve stimulation.* Neurosurgery, 2011. **69**(6): p. 1210-1217.
29. LoPresti, M.A., et al., *Pediatric Vagus Nerve Stimulation: Case Series Outcomes and Future Directions.* Neurosurgery, 2022: p. 10.1227.
30. Babajani-Feremi, A., et al., *Predicting seizure outcome of vagus nerve stimulation using MEG-based network topology.* NeuroImage: Clinical, 2018. **19**: p. 990-999.
31. Sangare, A., et al., *The effectiveness of vagus nerve stimulation in drug-resistant epilepsy correlates with vagus nerve stimulation-induced electroencephalography desynchronization.* Brain Connectivity, 2020. **10**(10): p. 566-577.
32. Mithani, K., et al., *Somatosensory evoked fields predict response to vagus nerve stimulation.* NeuroImage: Clinical, 2020. **26**: p. 102205.
33. Okamura, A., et al., *Secondary epileptogenesis on gradient magnetic-field topography correlates with seizure outcomes after vagus nerve stimulation.* Epilepsy Research, 2020. **167**: p. 106463.
34. Hilderink, J., et al., *Predicting success of vagus nerve stimulation (VNS) from EEG symmetry.* Seizure, 2017. **48**: p. 69-73.
35. De Vos, C., et al., *Predicting success of vagus nerve stimulation (VNS) from interictal EEG.* Seizure, 2011. **20**(7): p. 541-545.

# Supplementary Table 1 Results from EEG search:

Search performed on Pubmed on 15/01/24 as:

((Epilepsy[Title/Abstract]) OR (DRE[Title/Abstract])) AND (VNS[Title/Abstract]) AND ((EEG[Title/Abstract]) OR (Electroencephalography[Title/Abstract]) OR (Electroencephalogram[Title/Abstract])) AND ((prediction[Title/Abstract]) OR (predictor[Title/Abstract]) OR (predict[Title/Abstract]) OR (predictive[Title/Abstract]) OR (marker[Title/Abstract]) OR (biomarker[Title/Abstract]))

| **PMID** | **Title** | **Authors** | **DOI** |
| --- | --- | --- | --- |
| **31914344** | Can we predict response to vagus nerve stimulation in intractable epilepsy | Xiong J, Cao Y, Yang W, Chen Z, Yu Q. | 10.1080/00207454.2020.1713777 |
| **37914788** | Entropy in scalp EEG can be used as a preimplantation marker for VNS efficacy | Sklenarova B, Chladek J, Macek M, Brazdil M, Chrastina J, Jurkova T, Burilova P, Plesinger F, Zatloukalova E, Dolezalova I. | 10.1038/s41598-023-46113-z |
| **30173606** | The vagus afferent network: emerging role in translational connectomics | Hachem LD, Wong SM, Ibrahim GM. | 10.3171/2018.6.FOCUS18216 |
| **22367987** | Therapeutic devices for epilepsy | Fisher RS. | 10.1002/ana.22621 |
| **36136103** | Electroencephalogram and heart rate variability features as predictors of responsiveness to vagus nerve stimulation in patients with epilepsy: a systematic review | Madhani SI, Abbasi M, Liu Y, Larco JA, Nicolai E, Worrell G, Savastano L. | 10.1007/s00381-022-05653-x |
| **32840088** | [Application of scalp electroencephalogram in treatment of refractory epilepsy with vagus nerve stimulation] | Qin X, Yuan Y, Chen Y, Liao J, Lin S, Yang Z, Li L. | 10.7507/1001-5515.201909002 |
| **14747003** | Brain stimulation for epilepsy | Theodore WH, Fisher RS. | 10.1016/s1474-4422(03)00664-1 |
| **34668148** | Vagus Nerve Stimulation Elicits Sleep EEG Desynchronization and Network Changes in Responder Patients in Epilepsy | Vespa S, Heyse J, Stumpp L, Liberati G, Ferrao Santos S, Rooijakkers H, Nonclercq A, Mouraux A, van Mierlo P, El Tahry R. | 10.1007/s13311-021-01124-4 |
| **37928141** | Vagus nerve stimulation for treating developmental and epileptic encephalopathy in young children | Geng G, Hu W, Meng Y, Zhang H, Zhang H, Chen C, Zhang Y, Gao Z, Liu Y, Shi J. | 10.3389/fneur.2023.1191831 |
| **35386419** | Prediction of Vagal Nerve Stimulation Efficacy in Drug-Resistant Epilepsy (PRECISE): Prospective Study for Pre-implantation Prediction/Study Design | Dolezalova I, Koritakova E, Souckova L, Chrastina J, Chladek J, Stepanova R, Brazdil M. | 10.3389/fneur.2022.839163 |
| **36700739** | Pediatric Vagus Nerve Stimulation: Case Series Outcomes and Future Directions | LoPresti MA, Katlowitz KA, Sharma H, McGinnis JP, Weiner HL. | 10.1227/neu.0000000000002326 |
| **36039882** | Vagus nerve stimulation outcome prediction: from simple parameters to advanced models | Chrastina J, Novak Z, Zeman T, Dolezalova I, Zatloukalova E, Brazdil M. | 10.4149/BLL_2022_103 |
| **27714458** | An interictal EEG can predict the outcome of vagus nerve stimulation therapy for children with intractable epilepsy | Kim MJ, Yum MS, Kim EH, Lee YJ, Lee J, Hong S, You SJ, Hwang YS, Ko TS. | 10.1007/s00381-016-3261-5 |
| **37170486** | Brain functional connectivity-based prediction of vagus nerve stimulation efficacy in pediatric pharmacoresistant epilepsy | Chen H, Wang Y, Ji T, Jiang Y, Zhou XH. | 10.1111/cns.14257 |
| **37595607** | Functional brain connectivity indexes derived from low-density EEG of pre-implanted patients as VNS outcome predictors | Germany E, Teixeira I, Danthine V, Santalucia R, Cakiroglu I, Torres A, Verleysen M, Delbeke J, Nonclercq A, Tahry RE. | 10.1088/1741-2552/acf1cd |
| **35894770** | A prediction model integrating synchronization biomarkers and clinical features to identify responders to vagus nerve stimulation among pediatric patients with drug-resistant epilepsy | Ma J, Wang Z, Cheng T, Hu Y, Qin X, Wang W, Yu G, Liu Q, Ji T, Xie H, Zha D, Wang S, Yang Z, Liu X, Cai L, Jiang Y, Hao H, Wang J, Li L, Wu Y. | 10.1111/cns.13923 |
| **33836959** | Searching for a paradigm shift in the research on the epilepsies and associated neuropsychiatric comorbidities. From ancient historical knowledge to the challenge of contemporary systems complexity and emergent functions | Garcia-Cairasco N, Podolsky-Gondim G, Tejada J. | 10.1016/j.yebeh.2021.107930 |
| **31118916** | EEG Reactivity Predicts Individual Efficacy of Vagal Nerve Stimulation in Intractable Epileptics | Brázdil M, Doležalová I, Koritáková E, Chládek J, Roman R, Pail M, Jurák P, Shaw DJ, Chrastina J. | 10.3389/fneur.2019.00392 |
| **28431290** | Predicting success of vagus nerve stimulation (VNS) from EEG symmetry | Hilderink J, Tjepkema-Cloostermans MC, Geertsema A, Glastra-Zwiers J, de Vos CC. | 10.1016/j.seizure.2017.03.020 |
| **37575303** | Predictive factors for successful vagus nerve stimulation in patients with refractory epilepsy: real-life insights from a multicenter study | Pires do Prado HJ, Pinto LF, Bezerra DF, de Paola L, Arruda F, de Oliveira AJ, Romão TT, Lessa VCC, Silva JDS, D'Andrea-Meira I. | 10.3389/fnins.2023.1210221 |
| **32604021** | Long-term effects of vagus nerve stimulation in refractory pediatric epilepsy: A single-center experience | Yalnizoglu D, Ardicli D, Bilginer B, Konuskan B, Karli Oguz K, Akalan N, Turanli G, Saygi S, Topcu M. | 10.1016/j.yebeh.2020.107147 |
| **29940349** | Transcutaneous vagal nerve stimulatio (t-VNS): An adjunctive treatment option for refractory epilepsy | Barbella G, Cocco I, Freri E, Marotta G, Visani E, Franceschetti S, Casazza M. | 10.1016/j.seizure.2018.06.016 |
| **30802843** | Predictors of seizure reduction outcome after vagus nerve stimulation in drug-resistant epilepsy | Wang HJ, Tan G, Zhu LN, Chen D, Xu D, Chu SS, Liu L. | 10.1016/j.seizure.2019.02.010 |
| **21514181** | Predicting success of vagus nerve stimulation (VNS) from interictal EEG | de Vos CC, Melching L, van Schoonhoven J, Ardesch JJ, de Weerd AW, van Lambalgen HC, van Putten MJ. | 10.1016/j.seizure.2011.04.002 |
| **35806980** | Prediction of the Responsiveness to Vagus-Nerve Stimulation in Patients with Drug-Resistant Epilepsy via Directed-Transfer-Function Analysis of Their Perioperative Scalp EEGs | Kim D, Kim T, Hwang Y, Lee CY, Joo EY, Seo DW, Hong SB, Shon YM. | 10.3390/jcm11133695 |
| **33636552** | Pre-ictal heart rate variability alterations in focal onset seizures and response to vagus nerve stimulation | Hödl S, Olbert E, Mahringer C, Struhal W, Carrette E, Meurs A, Gadeyne S, Dauwe I, Goossens L, Raedt R, Boon P, Vonck K. | 10.1016/j.seizure.2021.02.017 |
| **34045942** | A Novel Statistical Model for Predicting the Efficacy of Vagal Nerve Stimulation in Patients With Epilepsy (Pre-X-Stim) Is Applicable to Different EEG Systems | Korit Áková E, Doležalová I, Chládek J, Jurková T, Chrastina J, Plešinger F, Roman R, Pail M, Jurák P, Shaw DJ, Brázdil M. | 10.3389/fnins.2021.635787 |
| **12521358** | Stimulation of the nervous system for the management of seizures: current and future developments | Murphy JV, Patil A. | 10.2165/00023210-200317020-00003 |
| **28414968** | Investigating the correlation between short-term effectiveness of VNS Therapy in reducing the severity of seizures and long-term responsiveness | Ravan M. | 10.1016/j.eplepsyres.2017.04.008 |
| **15716532** | Vagus nerve stimulation: predictors of seizure freedom | Janszky J, Hoppe M, Behne F, Tuxhorn I, Pannek HW, Ebner A. | 10.1136/jnnp.2004.037085 |
| **21323924** | Increased hippocampal noradrenaline is a biomarker for efficacy of vagus nerve stimulation in a limbic seizure model | Raedt R, Clinckers R, Mollet L, Vonck K, El Tahry R, Wyckhuys T, De Herdt V, Carrette E, Wadman W, Michotte Y, Smolders I, Boon P, Meurs A. | 10.1111/j.1471-4159.2011.07214.x |
| **37553557** | Transcutaneous auricular vagus nerve stimulation improves working memory in temporal lobe epilepsy: A randomized double-blind study | Pan L, Wang J, Wu W, Wang Y, Zhu Y, Song Y. | 10.1111/cns.14395 |
| **27450311** | Baseline elevation and reduction in cardiac electrical instability assessed by quantitative T-wave alternans in patients with drug-resistant epilepsy treated with vagus nerve stimulation in the AspireSR E-36 trial | Verrier RL, Nearing BD, Olin B, Boon P, Schachter SC. | 10.1016/j.yebeh.2016.06.016 |

# Supplementary Table 2 Results from MEG search:

Search performed on Pubmed on 15/01/24 as:

((Epilepsy[Title/Abstract]) OR (DRE[Title/Abstract])) AND (VNS[Title/Abstract]) AND ((MEG[Title/Abstract]) OR (Magnetoencephalography[Title/Abstract]) OR (Magnetoencephalogram[Title/Abstract])) AND ((prediction[Title/Abstract]) OR (predictor[Title/Abstract]) OR (predict[Title/Abstract]) OR (predictive[Title/Abstract]) OR (marker[Title/Abstract]) OR (biomarker[Title/Abstract]))

| **PMID** | **Title** | **Authors** | **DOI** |
| --- | --- | --- | --- |
| **32070812** | Somatosensory evoked fields predict response to vagus nerve stimulation | Mithani K, Wong SM, Mikhail M, Pourmotabbed H, Pang E, Sharma R, Yau I, Ochi A, Otsubo H, Snead OC, Donner E, Go C, Widjaja E, Babajani-Feremi A, Ibrahim GM. | 10.1016/j.nicl.2020.102205 |
| **31393626** | Connectomic Profiling Identifies Responders to Vagus Nerve Stimulation | Mithani K, Mikhail M, Morgan BR, Wong S, Weil AG, Deschenes S, Wang S, Bernal B, Guillen MR, Ochi A, Otsubo H, Yau I, Lo W, Pang E, Holowka S, Snead OC, Donner E, Rutka JT, Go C, Widjaja E, Ibrahim GM. | 10.1002/ana.25574 |
| **30003036** | Predicting seizure outcome of vagus nerve stimulation using MEG-based network topology | Babajani-Feremi A, Noorizadeh N, Mudigoudar B, Wheless JW. | 10.1016/j.nicl.2018.06.017 |

# Supplementary Table 3 Results from MRI search:

Search performed on Pubmed on 15/01/24 as:

((Epilepsy[Title/Abstract]) OR (DRE[Title/Abstract])) AND (VNS[Title/Abstract]) AND ((MRI[Title/Abstract]) OR (Magnetic Resonance Imaging[Title/Abstract]) OR (Structural[Title/Abstract])) AND ((prediction[Title/Abstract]) OR (predictor[Title/Abstract]) OR (predict[Title/Abstract]) OR (predictive[Title/Abstract]) OR (marker[Title/Abstract]) OR (biomarker[Title/Abstract]))

| **PMID** | **Title** | **Authors** | **DOI** |
| --- | --- | --- | --- |
| **32862454** | Biomarkers of seizure response to vagus nerve stimulation: A scoping review | Workewych AM, Arski ON, Mithani K, Ibrahim GM. | 10.1111/epi.16661 |
| **36415145** | Brain-clinical signatures for vagus nerve stimulation response | Guo Z, Mo J, Zhang C, Zhang J, Hu W, Zhang K. | 10.1111/cns.14021 |
| **30173606** | The vagus afferent network: emerging role in translational connectomics | Hachem LD, Wong SM, Ibrahim GM. | 10.3171/2018.6.FOCUS18216 |
| **36700739** | Pediatric Vagus Nerve Stimulation: Case Series Outcomes and Future Directions | LoPresti MA, Katlowitz KA, Sharma H, McGinnis JP, Weiner HL. | 10.1227/neu.0000000000002326 |
| **37928141** | Vagus nerve stimulation for treating developmental and epileptic encephalopathy in young children | Geng G, Hu W, Meng Y, Zhang H, Zhang H, Chen C, Zhang Y, Gao Z, Liu Y, Shi J. | 10.3389/fneur.2023.1191831 |
| **32717714** | Epilepsy duration as an independent predictor of response to vagus nerve stimulation | Zhu J, Xu C, Zhang X, Qiao L, Wang X, Zhang X, Yan X, Ni D, Yu T, Zhang G, Li Y. | 10.1016/j.eplepsyres.2020.106432 |
| **31393626** | Connectomic Profiling Identifies Responders to Vagus Nerve Stimulation | Mithani K, Mikhail M, Morgan BR, Wong S, Weil AG, Deschenes S, Wang S, Bernal B, Guillen MR, Ochi A, Otsubo H, Yau I, Lo W, Pang E, Holowka S, Snead OC, Donner E, Rutka JT, Go C, Widjaja E, Ibrahim GM. | 10.1002/ana.25574 |
| **36686529** | Vagus nerve stimulation for pharmacoresistant epilepsy secondary to encephalomalacia: A single-center retrospective study | Guo M, Wang J, Xiong Z, Deng J, Zhang J, Tang C, Kong X, Wang X, Guan Y, Zhou J, Zhai F, Luan G, Li T. | 10.3389/fneur.2022.1074997 |
| **33836959** | Searching for a paradigm shift in the research on the epilepsies and associated neuropsychiatric comorbidities. From ancient historical knowledge to the challenge of contemporary systems complexity and emergent functions | Garcia-Cairasco N, Podolsky-Gondim G, Tejada J. | 10.1016/j.yebeh.2021.107930 |
| **37595607** | Functional brain connectivity indexes derived from low-density EEG of pre-implanted patients as VNS outcome predictors | Germany E, Teixeira I, Danthine V, Santalucia R, Cakiroglu I, Torres A, Verleysen M, Delbeke J, Nonclercq A, Tahry RE. | 10.1088/1741-2552/acf1cd |
| **32745494** | The effect of vagal nerve stimulation on hippocampal-thalamic functional connectivity in epilepsy patients | Zhu J, Xu C, Zhang X, Qiao L, Wang X, Yan X, Ni D, Yu T, Zhang G, Li Y. | 10.1016/j.brainresbull.2020.07.023 |
| **26979179** | Combined surgical intervention with vagus nerve stimulation following corpus callosotomy in patients with Lennox-Gastaut syndrome | Katagiri M, Iida K, Kagawa K, Hashizume A, Ishikawa N, Hanaya R, Arita K, Kurisu K. | 10.1007/s00701-016-2765-9 |
| **15716532** | Vagus nerve stimulation: predictors of seizure freedom | Janszky J, Hoppe M, Behne F, Tuxhorn I, Pannek HW, Ebner A. | 10.1136/jnnp.2004.037085 |
| **29738985** | Single-center long-term results of vagus nerve stimulation for epilepsy: A 10-17 year follow-up study | Chrastina J, Novák Z, Zeman T, Kočvarová J, Pail M, Doležalová I, Jarkovský J, Brázdil M. | 10.1016/j.seizure.2018.04.022 |
| **23041031** | Long-term results of vagal nerve stimulation for adults with medication-resistant epilepsy who have been on unchanged antiepileptic medication | García-Navarrete E, Torres CV, Gallego I, Navas M, Pastor J, Sola RG. | 10.1016/j.seizure.2012.09.008 |
| **18355464** | [Long-term efficiency of vagus nerve stimulation (VNS) in non-surgical refractory epilepsies in adolescents and adults] | Montavont A, Demarquay G, Ryvlin P, Rabilloud M, Guénot M, Ostrowsky K, Isnard J, Fischer C, Mauguière F. | 10.1016/S0035-3787(07)78401-1 |

# Supplementary Table 4 Results from DWI search:

Search performed on Pubmed on 15/01/24 as:

((Epilepsy[Title/Abstract]) OR (DRE[Title/Abstract])) AND (VNS[Title/Abstract]) AND ((DWI[Title/Abstract]) OR (DTI[Title/Abstract]) OR (Diffusion weighted imaging[Title/Abstract]) OR (Diffusing tensor imaging)) AND ((prediction[Title/Abstract]) OR (predictor[Title/Abstract]) OR (predict[Title/Abstract]) OR (predictive[Title/Abstract]) OR (marker[Title/Abstract]) OR (biomarker[Title/Abstract]))

| **PMID** | **Title** | **Authors** | **DOI** |
| --- | --- | --- | --- |
| **31393626** | Connectomic Profiling Identifies Responders to Vagus Nerve Stimulation | Mithani K, Mikhail M, Morgan BR, Wong S, Weil AG, Deschenes S, Wang S, Bernal B, Guillen MR, Ochi A, Otsubo H, Yau I, Lo W, Pang E, Holowka S, Snead OC, Donner E, Rutka JT, Go C, Widjaja E, Ibrahim GM. | 10.1002/ana.25574 |
| **33836959** | Searching for a paradigm shift in the research on the epilepsies and associated neuropsychiatric comorbidities. From ancient historical knowledge to the challenge of contemporary systems complexity and emergent functions | Garcia-Cairasco N, Podolsky-Gondim G, Tejada J. | 10.1016/j.yebeh.2021.107930 |

# Supplementary Table 5 Results from fMRI search:

Search performed on Pubmed on 15/01/24 as:

((Epilepsy[Title/Abstract]) OR (DRE[Title/Abstract])) AND (VNS[Title/Abstract]) AND ((fMRI[Title/Abstract]) or (Functional MRI[Title/Abstract]))AND ((prediction[Title/Abstract]) OR (predictor[Title/Abstract]) OR (predict[Title/Abstract]) OR (predictive[Title/Abstract]) OR (marker[Title/Abstract]) OR (biomarker[Title/Abstract]))

| **PMID** | **Title** | **Authors** | **DOI** |
| --- | --- | --- | --- |
| **30173606** | The vagus afferent network: emerging role in translational connectomics | Hachem LD, Wong SM, Ibrahim GM. | 10.3171/2018.6.FOCUS18216 |
| **32745494** | The effect of vagal nerve stimulation on hippocampal-thalamic functional connectivity in epilepsy patients | Zhu J, Xu C, Zhang X, Qiao L, Wang X, Yan X, Ni D, Yu T, Zhang G, Li Y. | 10.1016/j.brainresbull.2020.07.023 |
| **28971013** | Presurgical thalamocortical connectivity is associated with response to vagus nerve stimulation in children with intractable epilepsy | Ibrahim GM, Sharma P, Hyslop A, Guillen MR, Morgan BR, Wong S, Abel TJ, Elkaim L, Cajigas I, Shah AH, Fallah A, Weil AG, Altman N, Bernal B, Medina S, Widjaja E, Jayakar P, Ragheb J, Bhatia S. | 10.1016/j.nicl.2017.09.015 |
